# *P1* Epigenetic Regulation in Leaves of High Altitude Maize Landraces: Effect of UV-B Radiation

**DOI:** 10.3389/fpls.2016.00523

**Published:** 2016-04-21

**Authors:** Sebastián P. Rius, Julia Emiliani, Paula Casati

**Affiliations:** Facultad de Ciencias Bioquímicas y Farmacéuticas, Centro de Estudios Fotosintéticos y Bioquímicos (CEFOBI-CONICET), Universidad Nacional de RosarioRosario, Argentina

**Keywords:** epigenetic regulation, flavonoids, maize, P1 transcription factor, UV-B

## Abstract

P1 is a R2R3-MYB transcription factor that regulates the accumulation of a specific group of flavonoids in maize floral tissues, such as flavones and phlobaphenes. P1 is also highly expressed in leaves of maize landraces adapted to high altitudes and higher levels of UV-B radiation. In this work, we analyzed the epigenetic regulation of the *P1* gene by UV-B in leaves of different maize landraces. Our results demonstrate that DNA methylation in the *P1* proximal promoter, intron1 and intron2 is decreased by UV-B in all lines analyzed; however, the basal DNA methylation levels are lower in the landraces than in B73, a low altitude inbred line. DNA demethylation by UV-B is accompanied by a decrease in H3 methylation at Lys 9 and 27, and by an increase in H3 acetylation. smRNAs complementary to specific regions of the proximal promoter and of intron 2 3′ end are also decreased by UV-B; interestingly, *P1* smRNA levels are lower in the landraces than in B73 both under control conditions and after UV-B exposure, suggesting that smRNAs regulate *P1* expression by UV-B in maize leaves. Finally, we investigated if different P1 targets in flower tissues are also regulated by this transcription factor in response to UV-B. Some targets analyzed show an induction in maize landraces in response to UV-B, with higher basal expression levels in the landraces than in B73; however, not all the transcripts analyzed were found to be regulated by UV-B in leaves.

## Introduction

Due to its sessile condition, plants continually adjust their growth and physiology to the changing environmental situations. In this context, epigenetic regulation seems to have a crucial role as a linker between the environment and the genome. Unlike genetic inheritance, the epigenetic modifications are unstable and are influenced by the environment (Baulcombe and Dean, 2014). Epigenetic mechanisms, such as methylation of DNA cytosine residues, covalent modifications of histones, ATP-dependent reorganization and positioning of DNA-histones and small RNAs (smRNAs), can modify chromatin structure and as a consequence, gene expression (Verbsky and Richards, 2001; Eberharter and Becker, 2002; Pfluger and Wagner, 2007; Vaillant and Paszkowski, 2007). DNA methylation and H3 methylation at K9 and K27 are generally associated with silent chromatin, while histone acetylation is usually linked to euchromatin (Vaillant and Paszkowski, 2007). Environmental stresses can modify epigenetic marks; these marks can be inherited as a preadaptation by subsequent generations (Pascual et al., 2014). For example, in *Nicotiana tabacum*, salt stress induces a significant DNA demethylation in genes encoding enzymes of the flavonoid pathway (Bharti et al., 2015). The expression of genes during different stress conditions was also reported to be under the regulation of chromatin-associated modifications (Henderson and Jacobsen, 2007). Moreover, epigenetic silencing can be an adaptation of plants to climates with different UV-B incidence levels (Questa et al., 2010).

In maize, *P1* encodes an R2R3-MYB transcription factor that regulates the accumulation of a specific group of flavonoids in maize floral tissues, the flavones and the phlobaphenes (Grotewold et al., 1994). P1 controls the accumulation of these pigments by activating the expression of a subset of maize flavonoid biosynthetic genes (Grotewold et al., 1994, 1998; Quattrocchio et al., 2006) and it is primarily expressed in floral tissues, including pericarps, cob glumes, silks and husks. Among the compounds controlled by *P1* are the flavones, important phytochemicals that protect against a number of maize pathogens, provide plants with UV shield and are significant nutraceutical components of the human diet (Quattrocchio et al., 2006). High-altitude maize landraces grown from 2000 to 3400 m naturally receive higher UV-B than plants at lower altitudes and similar latitudes (McKenzie et al., 2007). Previously, we demonstrated that some maize landraces adapted to high altitudes accumulate flavones and express *P1* in leaves and other green tissues in the presence of UV-B, in sharp departure to the floral-organ specific expression domain of *P1* found in most other maize inbred lines (Casati and Walbot, 2005; Rius et al., 2012). These results suggest that a large P1 allelic diversity may exist as a consequence of plants growing in diverse environments.

The *P1* gene is in general present in tandem repeats with different copy number depending on the genotype. The expansion of the *P1* expression domain in specific maize landraces that have adapted to high altitude (and hence to higher UV-B levels) could also be associated to changes in the molecular structure of the corresponding *P1* alleles. The number of *P1* copies varies depending on the maize genotype. The *P1-rr* (red pericarp and red cob) allele has been well characterized at the molecular level (Lechelt et al., 1989; Athma et al., 1992; Chinnusamy and Zhu, 2009) and contains a single coding sequence, which when expressed, confers pigmentation to both the kernel pericarp and the cob (Grotewold et al., 1991). Other allelic variants contain different *P1* copy numbers within their genomes. For example, the *P1-wr* (white pericarp and red cob) allele is composed of eleven gene copies arranged in a tandem head-to-tail array, and the B73 inbred line present a multiple copy *P1* cluster (Goettel and Messing, 2009). Also, landraces adapted to different altitudes have different *P1* copies (Rius et al., 2012). Comparison of the expression properties of the P1-*wr* and the P1-*rr* alleles has suggested that P1-*wr* is regulated both transcriptionally and post-transcriptionally in floral tissues (Chopra et al., 1996). The P1-*wr* gene has a tandemly amplified structure, and is hypermethylated compared to the single-copy P-*rr* allele. These results support a model of tissue-specific gene silencing that may be responsible for differences in expression between a single-copy and multicopy allele (Chopra et al., 1996). Functional analysis of the *P1-wr* promoter and coding sequences in transgenic maize plants, as well as studies of natural *P1* variants, have provided further support for the hypothesis that the organ-specific expression pattern of *P1-wr* is epigenetically regulated (Cocciolone et al., 2001); and studies have shown a correlation between H3K9me2 and DNA methylation in different P1 alleles (Chopra et al., 2003; Sekhon et al., 2007, 2012). Interestingly, a particular epigenetic state of the *P1-rr* allele is characterized by increased methylation of the *P1-rr* flanking regions and presents decreased levels of the *P1-rr* transcript (Sidorenko and Peterson, 2001). Also, plants with an spontaneous loss-of-function epimutation of the *P1-wr* allele shows a *P1-ww* phenotype (white kernel pericarps and white cob glumes); in these plants the level of cob pigmentation directly correlates with the degree of DNA demethylation in *P1* intron 2. Thus, distinct regulatory sequences in the *P1-wr* promoter and intron 2 regions can undergo independent epigenetic modifications to generate tissue-specific expression patterns (Sekhon et al., 2007).

In this work, we have analyzed the epigenetic regulation of the *P1* gene by UV-B in leaf tissues of two different high-altitude maize landraces and a low-altitude inbred line (B73), by analyzing changes in DNA methylation, histone modifications and smRNA levels after UV-B exposure. In addition, we investigated if different P1 targets in flower tissues are also regulated by this transcription factor in response to UV-B. Our results provide evidence suggesting that changes in the epigenetic state at specific *P1* regions are important in the regulation of flavonoid synthesis in maize leaves.

## Materials and methods

### Plant material and radiation treatments

*Zea mays* inbred line B73 and two high-altitude landraces Arrocillo Amarillo (Mexico), and Mishca (South American Andes) from altitudes between 2200 and 2800 m.a.s.l. were used. For the analysis of P1 targets, high altitude landraces Cacahuacintle (Mexico), Cónico Norteño (Mexico), and Confite Puneño (South American Andes) were also used. High altitude landraces were obtained from the Maize Genetics Cooperation Stock Center, University of Illinois, Urbana/Champaign National Plant Germplasm System (NPGS) (http://maizecoop.cropsci.uiuc.edu/USDA/ARS) and from the International Maize and Wheat Improvement Center (CIMMYT), D.F. Mexico, Mexico. The Instituto Nacional de Tecnología Agropecuaria (INTA, http://www.inta.gov.ar/) provided B73 seeds.

Plants were grown during 5 weeks in a greenhouse with a 16-h-light/8-h-dark photoperiod. UV-B was provided once for 8 h, starting 3 h after the beginning of the light period, using fixtures mounted 30 cm above the plants (Philips, F40UVB 40 W and TL 20 W/12) at a UV-B intensity of 2 W m^−2^, UV-A: 0.65 W m^−2^. The bulbs were covered with cellulose acetate to exclude wavelengths < 280 nm. As a control, plants were exposed for 8 h under the same lamps covered with polyester film (no UV-B treatment, UV-B: 0.04 W m^−2^, UV-A: 0.4 W m^−2^). Both groups of plants looked healthy after the treatments. Samples were pools of leaf pieces collected at the top of the canopy from multiple plants, to avoid plant-to-plant variability. Leaf samples were collected immediately after irradiation.

Lamp output was recorded using a UV-B/UV-A radiometer (UV203 A+B radiometer, Macam Photometrics, Ltd, Livingston, UK) to insure that both the bulbs and filters provided the designated UV dosage in all treatments.

### Chromatin immunoprecipitation (ChIP) experiments

For ChIP experiments, mature leaves from B73, Arrocillo and Mishca from plants irradiated with UV-B or kept under control conditions in the absence of UV-B were used. ChIP experiments were carried out as described in Casati et al. (2008). For each reaction, 4 μL of the following commercial antibodies were used: anti-H3 (dimethylated K9) (ab1220), anti-H3 (trimethylated K27) (ab6002), anti-H3 (ab1791) (Abcam, Cambridge, MA); and anti-N-terminal acetylated H3 (06-599, Upstate Biotechnology, Lake Placid, NY). All these antibodies were previously tested for cross reactivity against maize proteins (Casati et al., 2008; Questa et al., 2010). Three biological replicates from each genotype/treatment sample type were performed, and three qPCR experiments were done with each sample.

### RNA isolation, reverse transcription reaction and qRT-PCR

RNA samples were isolated using Trizol (Invitrogen, Carlsbad, CA) as described by Casati and Walbot (2003). RNA was isolated from a pool of top leaves (which received the greatest UV-B exposure) from six plants. Five micro gram of total RNA from each genotype/treatment combination were used for cDNA synthesis using Superscript II reverse transcriptase (Invitrogen).

cDNA was used as a template for quantitative PCR amplification in a MiniOPTICON2 apparatus (Bio-Rad), using the intercalation dye SYBRGreen I (Invitrogen) as a fluorescent reporter and Platinum Taq Polymerase (Invitrogen). Primers were designed to generate unique 150–250 bp-fragments using the PRIMER3 software (Rozen and Skaletsky, 2000). Three replicates were performed for each sample plus a negative control (reaction without reverse transcriptase). To normalize the data, primers for a *thioredoxin-like* transcript (AW927774) were used; this transcript is not regulated by UV-B. Primers used are listed in Table 1. Amplification conditions were as follows: 2 min denaturation at 94°C 40–45 cycles at94°C for 15 s, 57°C for 20 s, and 72°C for 20 s, followed by 10 min at 72°C. Melting curves for each PCR were determined by measuring the decrease of fluorescence with increasing temperature (from 65 to 98°C). To confirm the size of the PCR products and to check that they corresponded to a unique and expected product, the final products were separated on a 2% (w/v) agarose gel.

**Table 1:** **Primers and probe sequences used**.

**Primer/Probe name**	**Sequence 5′ – 3′**
EP5-8-F	ACGCGCGACCAGCTGCTAACCGTG
P1007ATG-R	TCGCAGCACGGCGCCCTCCCCAT
Ex1end-F	GTCGCTGCCCAAGAATGCAG
EP3-13-R	TCCGCCCGAAGGTAGTTGATCC
10908-F	GTCCTGTCCATTTCGCTTTG
11202 R	CGGCGTGTGTTTATATATGG
U6 probe	TCATCCTTGCGCAGGGGCCA
smRNA-P1 probe4 R	GCGTGGCGTCGACGTGGAACCGAGCTCGGC
smRNA-P1 probe5 R	TGCAGTTTTGGACCCTTTGCTCGGCGCCATAGGCTAT
smRNA-P1 probe6 R	CCTCTCACCGTCCGCAGTGTCAACGTTAAG GCGGGGGAAATCATTAGGGAGGGCCGGC
Thioredoxin-like F	GGACCAGAAGATTGCAGAAG
Thioredoxin-like R	CAGCATAGACAGGAGCAATG
EP3-13	TGGAGGTCGCTGCCCAAGAAT
P1-L010	TCCGCCCGAAGGTAGTTGATCC
STRS1-F	CAGGTGCTTGACATCTTGGA
STRS1-R	AGCTTCCCGTCTTTCTCCTC
JAC1-F	CCTGCTCTAAAACCGACGAC
JAC1-R	GCCTTCCCATTCTGTTGATG
WHP-F	GACCCGACCTACGAATAATG
WHP-R	AACTTGAGCTGGTGCACTG
MATE-F	CTCTGCCTCGAGACCTGGTA
MATE-R	GACAGAGAGTGAGGCCAAGG
LAC10-F	GGTAGCCGTTTTCATCGTAGAG
LAC10-R	AGGCCTCCTTTGTTAGATCCAC
F2H-F	CGGTCCATCCAAATTCAG
F2H-R	ACCAACATCGAACGGGTA
RHM-F	ACTTTACTTTTGGGCTGTCG
RHM-R	TCGACCTCCTCTGCTGTTC
CCR-F	CTCCTGCTCCTGTCCTACCAG
CCR-R	CCTCCTCTCACCTTGTTCAG
PAL1-F	GTCTCGACTCTCCACACCAC
PAL1-R	GGAGAGAACCAGCAGCAGTG
PAL2-F	GCCTCAGTGCCTCACCTAAG
PAL2-R	GGGCGAGGCGGTTATATAGG
PAL3-F	GGCTGCCATCCTATCCTATCC
PAL3-R	CCACCACTCACCTTGCTACAG
UGT1-F	GAGGAGCAGATTCGGTGAGC
UGT1-R	CGACTGACGACAGTGTCTGG
UGT3-F	ACTGGGCCTAGGCTAGACTGC
UGT3-R	GACCACCACAGTGGGGTATG
UGT4-F	AGTACGGCCATCCTCTGGTC
UGT4-R	CACCTTGACTCCGACCTTCC
UGT5-F	TGATTTCTGCGAGCCTGT
UGT5-R	AAGAAACGAGTGCGTGGA

### Enzyme digestion and quantitative PCR

The isolation of DNA templates for DNA methylation analysis by qPCR, and the analysis of the percentage of methylation was done as previously described (Oakes et al., 2006). One micro gram of DNA was used for each DNA digestion reaction. DNA was diluted to 50 ng μl^−1^ and digested overnight in a volume of 50 μl with 5 units of *Hpa*II or *Msp*I, or no enzyme as a control. After digestion, each PCR template was diluted 8-fold in water and incubated at 65°C for 30 min to inactivate the enzyme prior to the reaction assay. Three biological replicates were performed for each sample, and three qPCR experiments were done with each sample using specific primers flanking regions of interest (see Table 2). *Hpa*II digest the sequence CCGG but it does not cut the methylated form of this sequence, C^5*m*^CGG. If the CpG island does not contain C^5*m*^CGG, DNA is digested and no PCR product can be detected after amplification. As individual sample controls, PCR amplification was done on undigested DNA and DNA digested with the restriction enzyme *Msp*I using the above protocol. *Msp*I cuts both the unmethylated and methylated sequence; thus, no PCR product should be detected. The total amount of DNA from each sample was calculated using the standard curve derived from the non–restriction enzyme treated control. The methylation index was calculated as: (amount of *Hpa*II digested DNA/amount of input DNA)—(amount of *Msp*I digested DNA / amount of input DNA) multiplied by 100 [i.e., (methylated DNA—nonspecifically amplified DNA) × 100] to adjust for incomplete digestion (Bastian et al., 2005; Hashimoto et al., 2007).

**Table 2:** *****Hpa***II/***Msp***I restriction sites number and ***P1*** copy number**.

	**Proximal promoter**	**Intron 1**	**Intron 2**	***P1* copy number**
B73	7	4	3	14
ARR	7	4	3	1
MIS	7	4	3	8

*The sequences corresponding to the P1 proximal promoter, introns 1 and 2 are detailed in Figure S2*.

### Statistical analysis

Data presented were analyzed using one-way analysis of variance (ANOVA). Minimum significant differences were calculated by the Bonferroni, Tukey, Dunett, and Duncan tests (*P* < 0.05) using the SigmaStat Package.

### Northern blotting for smRNA detection

Northern blot assays were performed with 30 μg of RNA. For smRNA detection, total RNA was separated by electrophoresis in 15% polyacrylamide gels containing 7 M urea in Tris-borate-EDTA buffer (pH 8.0). Gels were transferred to a Hybond-NX membrane (Amersham) and were then UV cross-linked (1.200 μJ; Stratalinker; Stratagene). All probes were labeled at their 5′ end with the T4 Polynucleotide Kinase (Fermentas) in the presence of [γ-^32^P]ATP, and ^32^P-labeled oligonucleotides were separated from unreacted [γ-^32^P]ATP by chromatography through a small Sephadex® G-50 column in 10 mM Tris-HCl (pH 7.5) and 0.1% SDS. Mixed probes were hybridized to one piece of membrane. The specific radioactivity of the ^32^P-labeled oligonucleotides was 3 × 10^6^ cpm/pmol. Probes are listed in Table 1. U6 mRNA was used as a control for equal loading of RNA in each lane.

## Results

### UV-B induces changes in *P1* DNA methylation

Previously, we reported that P1, a transcription factor that regulates the expression of several genes that encode enzymes in the flavonoid pathway in maize, is highly expressed in leaves of high altitude maize landraces; and it is also regulated by UV-B radiation in these tissues (Casati and Walbot, 2005; Rius et al., 2012). Figure S1 shows a qRT-PCR analysis of *P1* expression in leaves of B73 and two high altitude landraces, Arrocillo Amarillo and Mishca, under control and UV-B conditions. The results again demonstrate that *P1* in expressed in maize leaves and that its expression is highly induced by UV-B radiation in the landraces.

Thus, and because *P1* expression pattern was previously correlated with epigenetic changes in the gene (Chopra et al., 2003; Sekhon et al., 2007, 2012); the first aim of this study was to determine if *P1* DNA methylation is changed by UV-B radiation, and whether this is true in a low altitude inbred, B73, and/or the two high altitude landraces, Arrocillo Amarillo and Mishca. To quantify methylation changes, a sensitive PCR protocol was used to detect both methylated and unmethylated sequences. Methylation levels from different *P1* sequences corresponding to the proximal promoter, intron 1 and intron 2 regions were quantified using qPCR with *Hpa*II and *Msp*I -digested DNA from control and UV-B treated plants. *Hpa*II and *Msp*I are isoschizomers that recognize the C/CGG site. However, *Hpa*II is unable to digest DNA when the internal cytosine is methylated, while *Msp*I digests DNA independently of cytosine methylation (see Materials and Methods). Table 1 and Figure S2 show the number of restriction sites recognized by these enzymes in each *P1* region.

**Figure 1:**
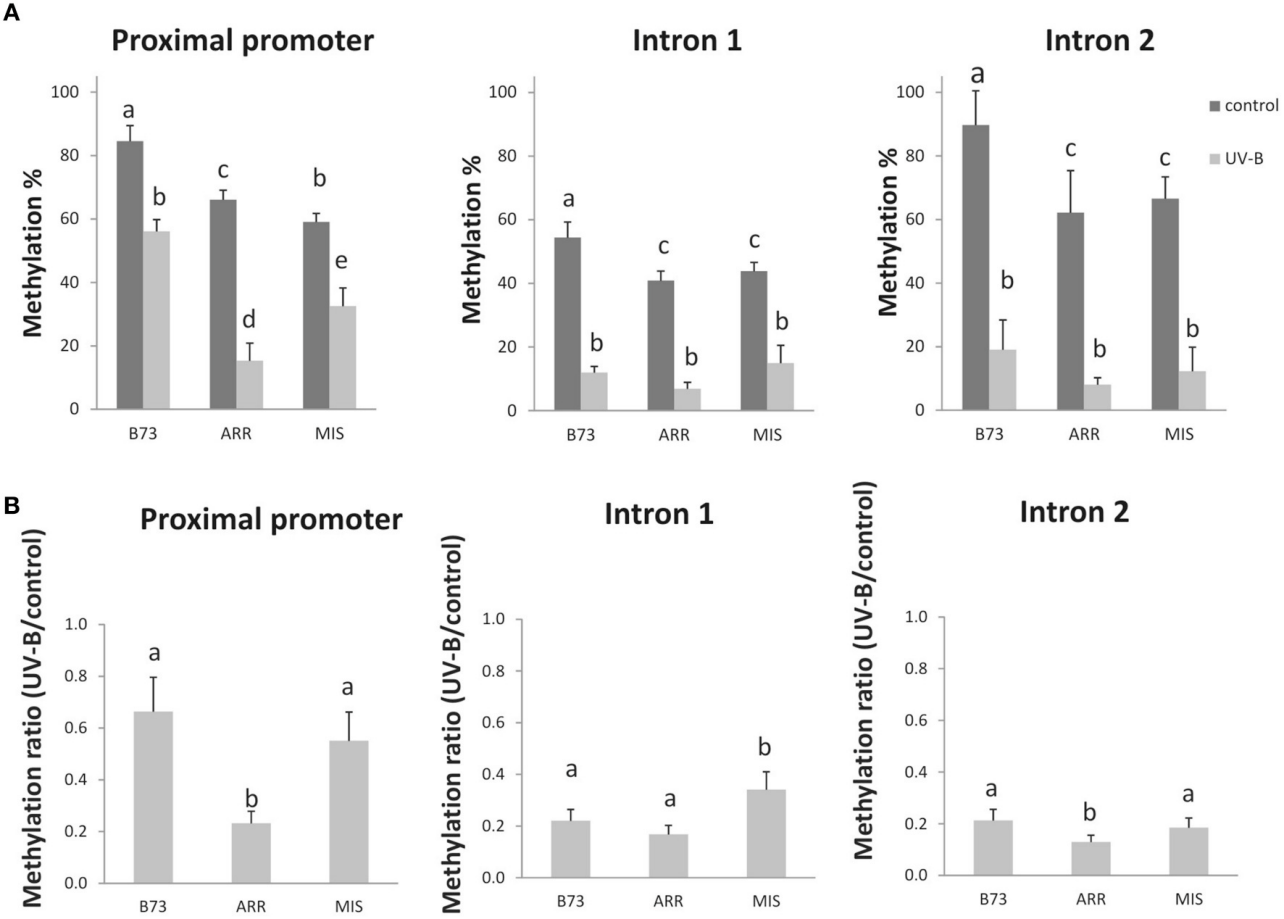
**DNA methylation changes in three regions of the ***P1*** gene in the B73 inbred line and Arrocillo (ARR) and Mishca (MIS) landraces after 8 h of UV-B exposure**. Methylation percentage measured by qPCR of digested DNA from the *P1* proximal promoter, introns 1 and 2 under control conditions and after UV-B exposure with *Hpa*II/*Msp*I restriction enzymes **(A)**. Ratio of DNA methylation in UV-B exposed plants vs. DNA methylation in control plants **(B)**. Primers were designed to amplify across the restriction sites (Table 1); therefore, amplification is expected if DNA is methylated and not digested. Three biological replicates were performed for each sample, and three qPCR experiments were done with each sample. Error bars are standard errors. Statistical significance was analyzed using ANOVA, Tukey test with *P* < 0.05; differences from the control are marked with different letters.

In all lines, the UV-B treatment induced a decrease in the percentage of DNA methylation in the three different regions analyzed. However, both under control conditions and after UV-B exposure, the *P1* promoter from the B73 inbred line presented a higher degree of methylation than that from the landraces. This was also true only under control conditions for intron 2. After UV-B exposure, DNA methylation in the *P1* promoter of the B73 line was similar to levels in the DNA of the landraces under control conditions in the absence of UV-B (Figure 1A). Interestingly, the demethylation in the promoter region and in intron 2 is significantly higher in Arrocillo than in the other two lines (Figure 1B). This could be associated to the different *P1* copy number in their genomes. Arrocillo contains only one *P1* copy, while B73 and Mishca have 13 and 8 copies, respectively (Table 2). However, methylation changes by UV-B were similar for the 3 lines analyzed for intron 1; with a small although still significant lower decrease measured in Mishca (Figure 1B). Thus, if there is any effect of *P1* copy number in DNA demethylation after UV-B exposure, this is only true for the promoter region. For both intron regions, UV-B radiation induced a decrease in DNA methylation of about 20% after 8 h of exposure, independently of the maize line. Under control conditions, intron 1 was partially methylated (between 40 and 50% methylation) in the three lines; while intron 2 was almost fully methylated in B73 but only around 60% in the landraces.

### Changes in H3 methylation by UV-B are associated with specific *P1* regions

Histone methylation in H3K9me2 and H3K27me3 is an epigenetic mark that mediates gene silencing and usually occurs in association with DNA methylation. Thus, we next investigated if the differences in DNA methylation observed in the different maize lines and after UV-B exposure were also accompanied by changes in the H3 methylation associated to the same DNA regions.

**Figure 2:**
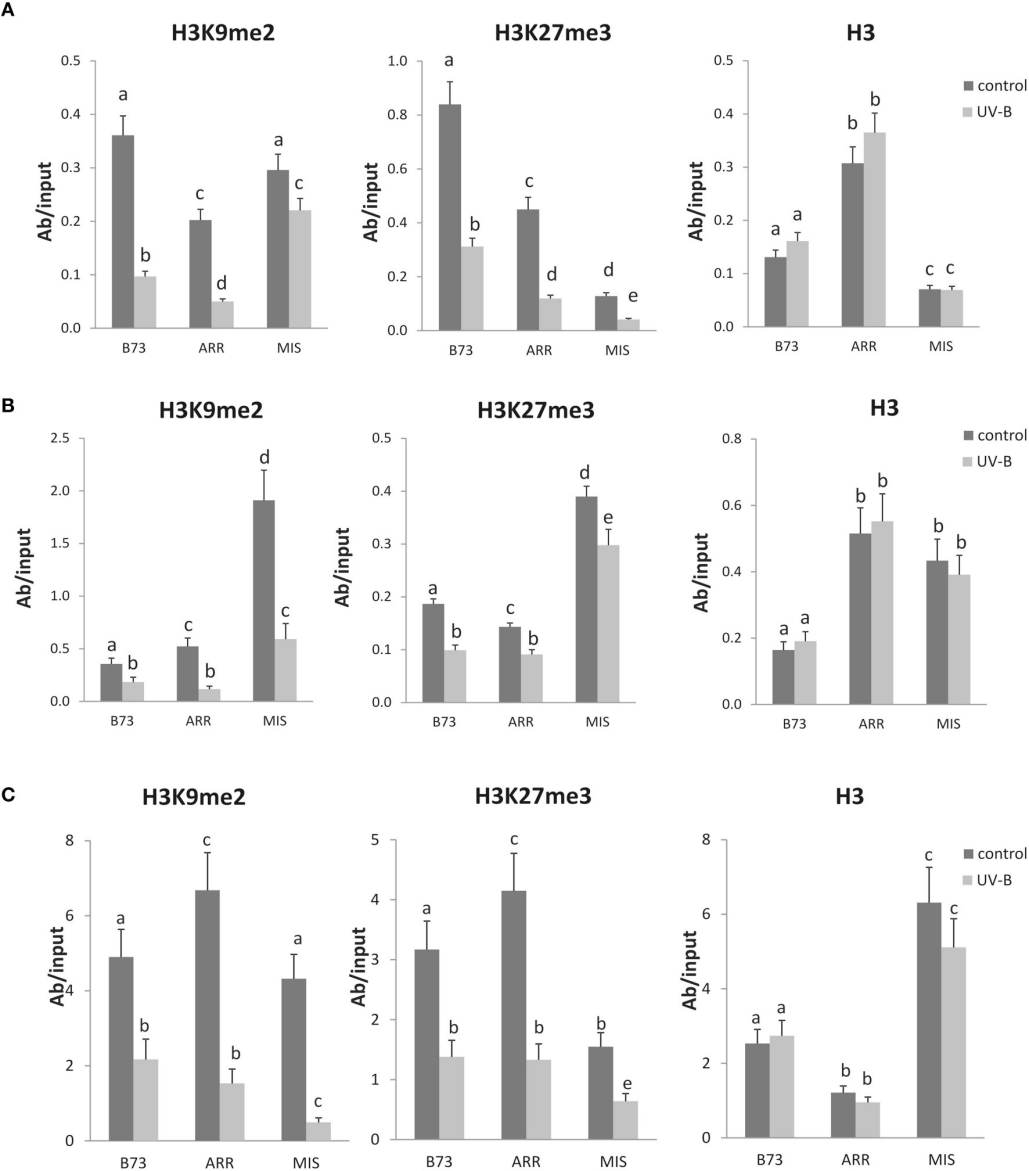
**Methylation state of H3 K9 and K27 associated with the ***P1*** proximal promoter, introns 1 and 2 of B73, Arrocillo (ARR), and Mishca (MIS)**. ChIP assays were done using antibodies specific for H3K9me2, H3K27me3, or total H3 in nuclei from leaves after an 8-h-UV-B treatment (UV-B) or kept under control conditions in the absence of UV-B (control). The immunoprecipitates were analyzed for the presence of the *P1* proximal promoter **(A)**, intron 1 **(B)**, and intron2 **(C)** sequences by qPCR. Enriched fractions from UV-B treated vs. control plants were compared. ChIP data were normalized to input DNA before immunoprecipitation. The signal detected in samples incubated in the absence of any antibody as a control was less than 5% of the signal when antibodies were used. Error bars are standard errors. Statistical significance was analyzed using ANOVA, Tukey test with *P* < 0.05; differences from the control are marked with different letters. Three biological replicates of chromatin immunoprecipitation (ChIP) were performed from each genotype/treatment sample type, and three qPCR experiments were done with each sample.

ChIP (chromatin immunoprecipitation) analyses were done using commercial antibodies against H3K9me2, H3K27me3, and total H3. B73 and the two landraces under control and after UV-B exposure were sampled, and the enrichment after immunoprecipitation was analyzed by qPCR using specific primers that amplify the *P1* proximal promoter, intron 1 and intron 2 (Table 1 and Figure 2). The coding region of a constitutive *thioredoxin-like* gene was used as an internal control to show that there were no significant changes in the enriched fractions for the control gene with either antibody (Figure S3). To evaluate nonspecific binding, a qPCR reaction was done with samples incubated without any antibody as a control; all ChIPed samples were also analyzed in parallel with total DNA from sonicated nuclei to evaluate the selective recovery of gene segments. The percentage of DNA recovered relative to the DNA input when experiments were done in the absence of antibodies was always lower than five percent of the DNA recovered when antibodies were used.

The results presented in Figure 2A show that there is a decreased association of H3K9me2 with the *P1* proximal promoter of B73 and Arrocillo after UV-B exposure; in contrast, no significant changes in the enrichment of H3K9me2 are observed in Mishca samples after UV-B exposure.

**Figure 3:**
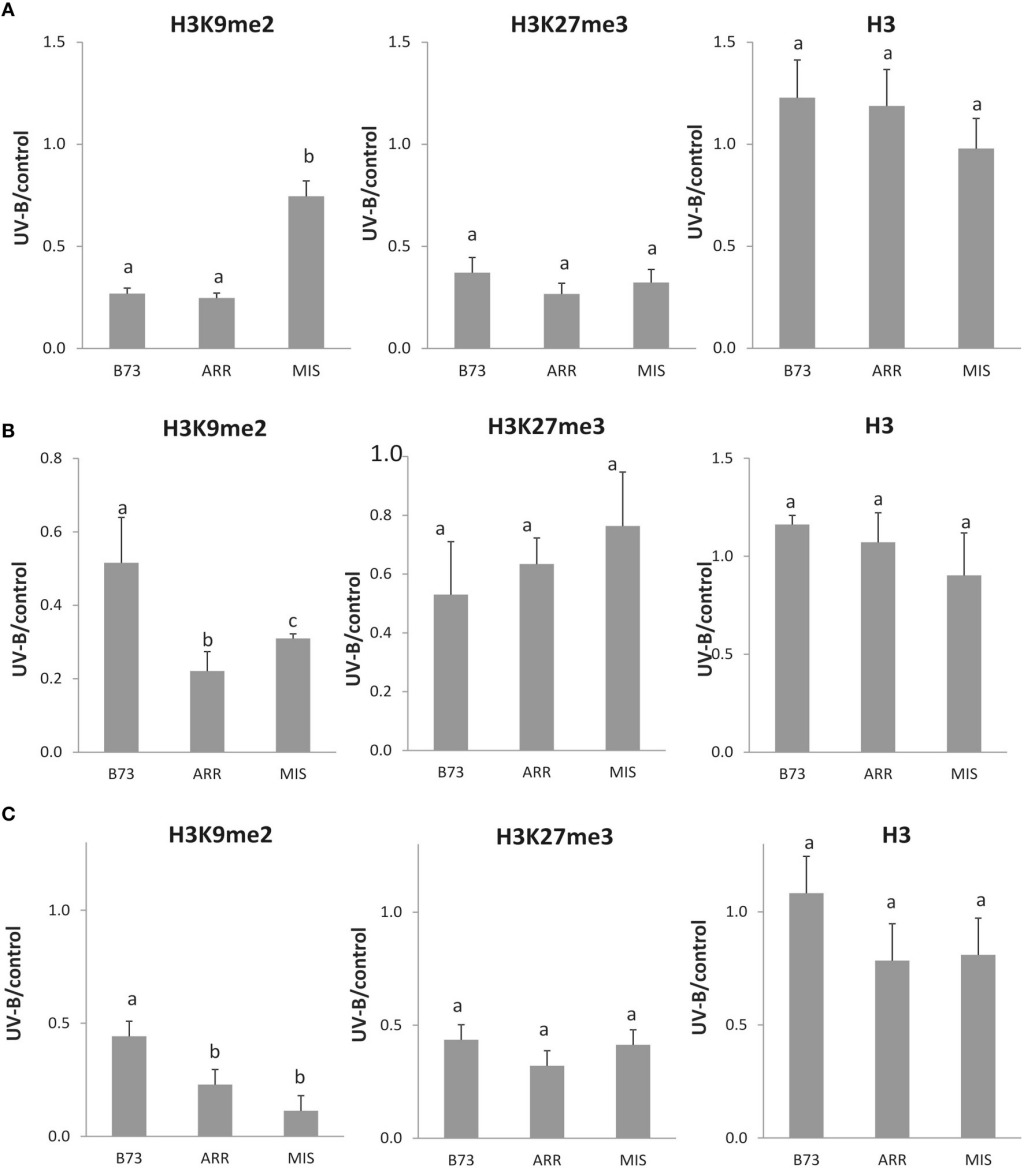
**Methylation state ratio of H3 K9 and K27 associated with the ***P1*** proximal promoter, introns 1 and 2 of B73, Arrocillo (ARR) and Mishca (MIS) enriched fractions from UV-B treated vs. control conditions plants**. *P1* proximal promoter **(A)**, intron 1 **(B)**, and intron 2 **(C)** sequences were analyzed. ChIP data were normalized to input DNA before immunoprecipitation. Absolute values correspond to data shown in Figure 2. The signal detected in samples incubated in the absence of any antibody as a control was less than 5% of the signal when antibodies were used. Error bars are standard errors. Statistical significance was analyzed using ANOVA, Tukey test with *P* < 0.05; differences from the control are marked with different letters. Three biological replicates of chromatin immunoprecipitation (ChIP) were performed from each genotype/treatment sample type, and three qPCR experiments were done with each sample.

On the other hand, when H3K27me3 associated to the *P1* proximal promoter region was analyzed, a significant decrease in enrichment was measured after the UV-B treatment in all three maize lines, which was of more than 50% in all lines (Figure 3A). In this case, H3K27me3 association to the B73 *P1* promoter was higher than that to Arrocillo and Mishca promoters, both under control conditions and after UV-B exposure (2-fold higher than Arrocillo and 8-fold higher than Mishca, Figure 2A). The 3 lines showed a similar decrease in H3K27me3 association with the proximal promoter after UV-B exposure (Figure 3A).

As a control, immunoprecipitation was also done using antibodies against total H3. No difference in enrichment was detected between control and UV-B treated samples for either B73 or landraces samples, although enrichment levels were different between lines (Figure 2A).

When a similar analysis was done for intron 1, a significant decrease in the enrichment of H3K9me2 associated to this intron was measured after the UV-B treatment in all lines. Despite this, H3K9me2 association to intron 1 was higher in Mishca than in B73 and Arrocillo (Figure 2B); however, both Mishca and Arrocillo showed a significant higher decrease in H3K9me2 enrichment than B73 plants after UV-B exposure (Figure 3B).

For H3K27me3, there was also a decreased association with intron 1 after UV-B exposure in all lines, although H3K27me3 association to intron 1 was higher in Mishca than in B73 and Arrocillo, similarly as measured for H3K9me2 (Figure 2B). In all lines, the relative decrease in H3K27me3 after the treatment was similar (Figure 3B). No difference in enrichment was detected in H3 binding to intron 1 between control and UV-B treated samples for either B73 or landraces samples, although enrichment levels were different between lines (Figure 2B).

**Figure 4:**
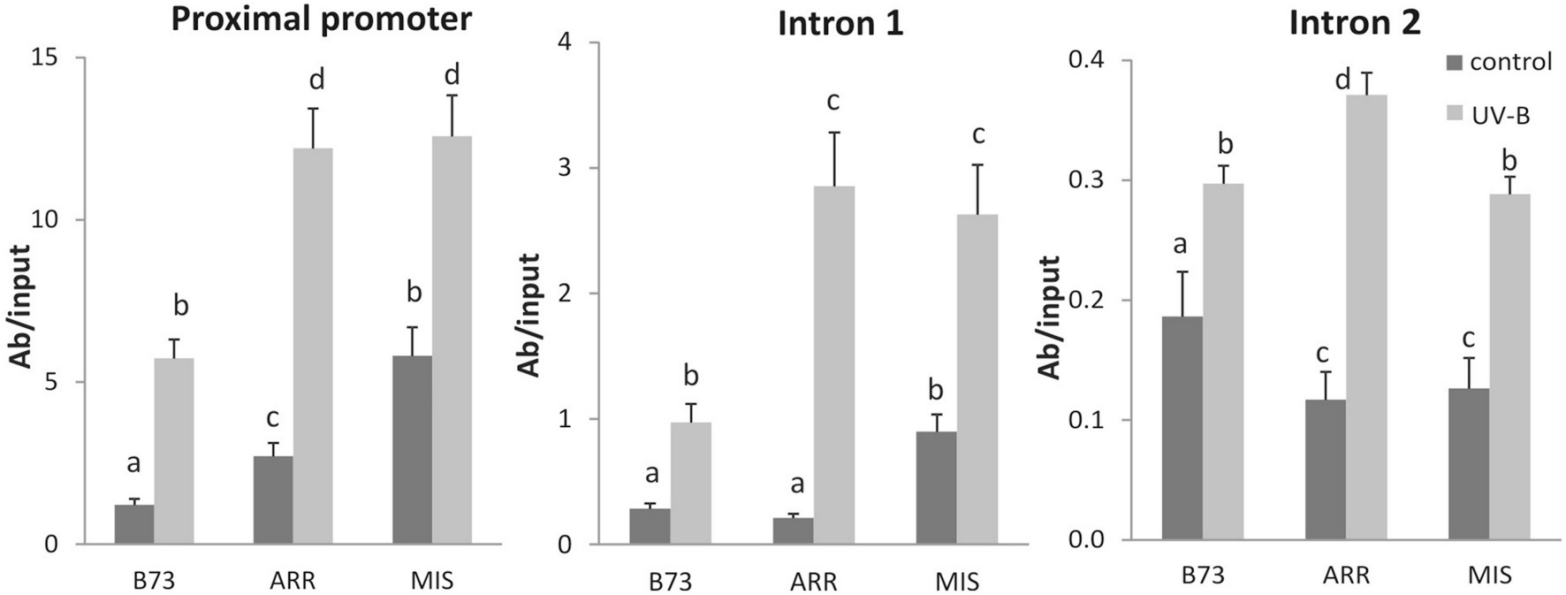
**Acetylation state of H3 associated with ***P1*** regions of B73, Arrocillo (ARR) and Mishca (MIS)**. ChIP assays were done using antibodies specific for N-terminal acetylated H3, in nuclei from leaves after an 8-h-UVB treatment (UV-B) or kept under control conditions in the absence of UV-B (control). The immunoprecipitates were analyzed for the presence of *P1* sequences of the proximal promoter, introns 1 and 2. Enriched fractions from UV-B treated vs. control plants were compared. ChIP data were normalized to input DNA before immunoprecipitation. The signal detected in samples incubated in the absence of any antibody as a control was less than 5% of the signal when antibodies were used. Error bars are standard errors. Statistical significance was analyzed using ANOVA, Tukey test with *P* < 0.05; differences from the control are marked with different letters. Three biological replicates of chromatin immunoprecipitation (ChIP) were performed from each genotype/treatment sample type, and three qPCR experiments were done with each sample.

Finally, when intron 2 was analyzed, both H3K9me2 and H3K27me3 enrichments were significantly decreased after the UV-B treatment in the 3 lines studied (Figure 4). For H3K9me2, although enrichment under control conditions was similar for the 3 lines, after UV-B exposure there was a higher decrease in histone methylation in the landraces than in B73 (Figure 3C). On the contrary, the decrease in H3K27me3 enrichment by UV-B was similar in the three lines (Figure 3C); although basal levels of enrichment in Mishca were significantly lower than in B73 and Arrocillo (Figure 2C). Again, no difference in enrichment was detected in H3 binding to intron 2 in control and UV-B treated samples from either B73 or the landraces, although enrichment levels were different between lines (Figure 2C).

Together, a decrease in the degree of association to H3K9me2 and H3K27me3 by UV-B occurs in the three *P1* regions analyzed in the lines under study. For H3K9me2, both intron regions showed a higher decrease in enrichment in the landraces than in B73 after the UV-B treatment; while the decrease in association to H3K27me3 was similar for the three lines. On the contrary, for the proximal promoter region, H3K27me3 association was lower in the landraces than in B73, both under control conditions and after UV-B exposure.

### H3 acetylation in *P1* regulatory regions is increased after UV-B exposure

Increases in transcript abundances often correlate with increased histone acetylation (Eberharter and Becker, 2002; Li et al., 2007). Thus, ChIP analysis was also performed using antibodies specific for acetylated Lys residues in the N-terminal tail of histone H3 (AcH3) to investigate if histone acetylation is also involved in the regulation of *P1* expression in maize leaves in response to UV-B.

*P1* proximal promoter, intron 1 and intron 2 regions were enriched in immunoprecipitated fractions using anti-AcH3 antibodies in control samples and after UV-B irradiation (Figure 4). Both under control conditions and after UV-B exposure, enrichment of AcH3 to the *P1* proximal promoter of both landraces was higher than that of B73. A similar result was observed when AcH3 association to the intron 1 region was analyzed. However, for intron 2, B73 showed higher H3 acetylation levels than the landraces under control conditions, while after the UV-B treatment H3 acetylation was similar in the three lines (Figure 4).

### Small RNAs could guide *P1* methylation after UV-B exposure

**Figure 5:**
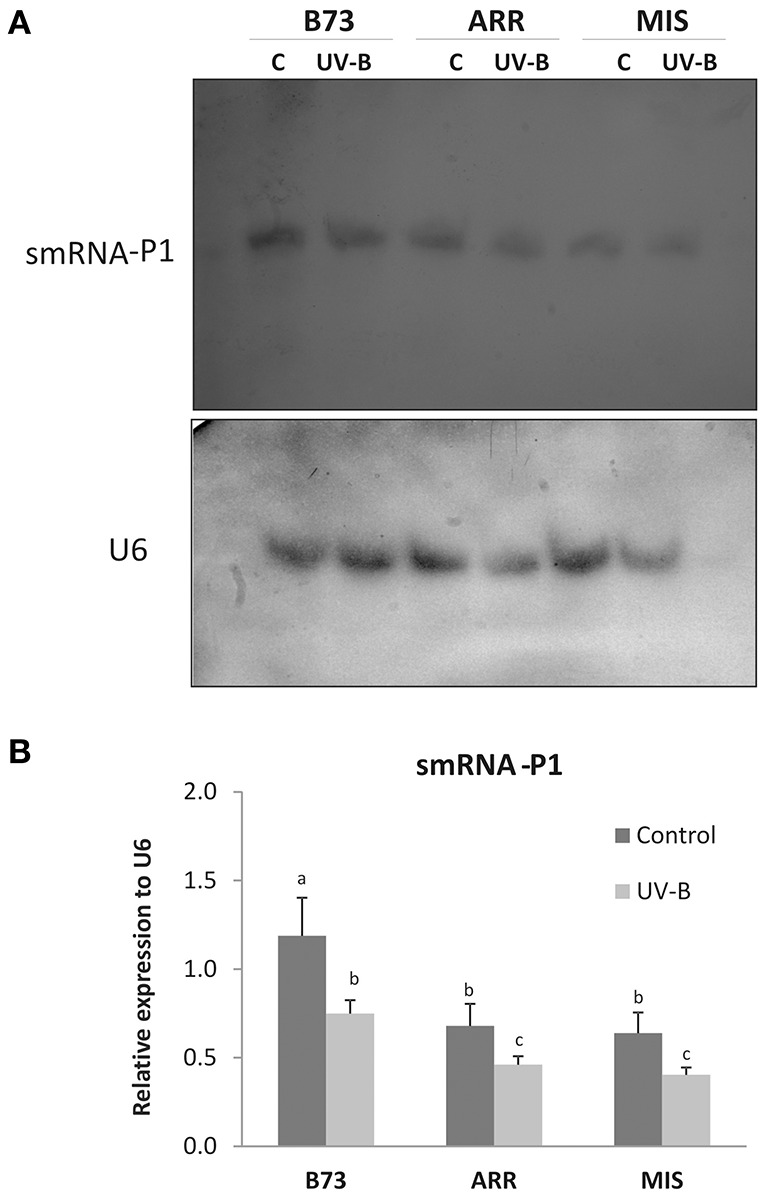
**Expression of smRNAs complementary to the ***P1*** proximal promoter and the 3′ end of intron 2 in leaves of B73, Arrocillo (ARR) and Mishca (MIS)**. Plants were irradiated with UV-B for 8 h or kept under control conditions in the absence of UV-B. **(A)** Northern blot analysis developed using ^32^P-labeled smRNA probes complementary to the *P1* proximal promoter (probes 4 and 5) and the 3′ end of intron 2 (probe 6), described in **Table 3**, or alternatively with an specific U6 probe. U6 mRNA was used as a control of equal loading of RNA in each lane. Each blot is representative of three individual experiments. **(B)** Densitometry analysis of replicated experiments normalized to U6. Different letters indicate significant differences between samples from control and UV-B irradiated plants (*p* < 0.05).

Tandem repeat sequences are frequently associated with gene silencing. The *P1* gene is in general present in tandem repeats with different copy number depending of the genotype, with some exceptions like Arrocillo, with only one *P1* copy in its genome. Tandem repeats throughout the genome can produce smRNAs, suggesting that repeat acquisition may be a general mechanism for the evolution of gene silencing. Interestingly, smRNAs play important roles in plants under stress conditions (Lin et al., 2013). To investigate whether changes in DNA methylation and histone methylation in *P1* regions by UV-B are correlated with smRNA production, smRNA levels that correspond to different regions of the *P1* gene (Rius et al., 2012) were measured by northern blot assays as shown in Figure 5 and Figure S4 in leaves of the different maize lines after UV-B exposure and in control conditions in the absence of UV-B.

While all the leaf samples expressed the population of smRNAs analyzed, the expression levels differed between control and treated plants, and also between B73 and the landraces at same treatment condition (Figure 5). Under control conditions, *P1* smRNAs in B73 leaves were twice as high as in the landraces. After 8 h of UV-B exposure, smRNAs complementary to discrete P1 sequences were decreased in all lines; however, smRNA levels were lower in the two landraces (Figure 5 and Figure S4).

Interestingly, *P1* copy number seems not to be related with expression levels of the selected smRNAs. While B73 and Mishca have high *P1* copy number, Arrocillo has only one copy. Thus, the smRNA levels seem to be independent of *P1* gene copy number but related to the genome background.

### Some but not all P1 target genes in pericarps and silks are regulated by UV-B in leaves

Finally, by RT-qPCR, we analyzed if a subset of genes previously identified as direct P1 targets in silks and pericarps (Morohashi et al., 2012) were also expressed in leaves of B73 and high altitude landraces, and were regulated by UV-B in correlation to *P1* expression patterns and epigenetic regulation in leaves.

**Figure 6:**
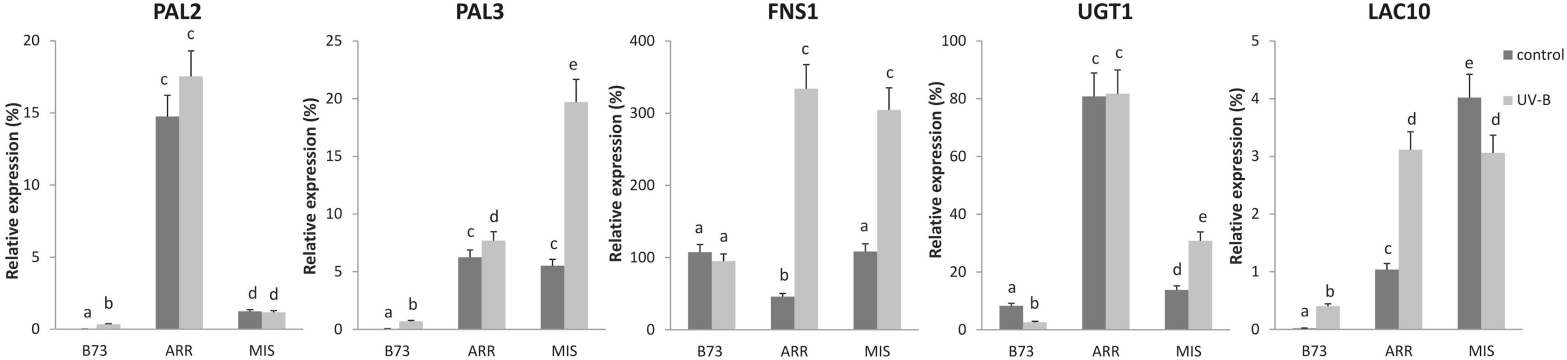
**Expression analysis of P1 target genes under control conditions and after UV-B exposure in leaves of B73, Arrocillo (ARR) and Mishca (MIS)**. Transcript levels relative to the reference *thr-like* gene that is not regulated by UV-B are shown. Statistical significance was analyzed using ANOVA, for each sample analyzed, different letters indicate significant differences with *P* < 0.05.

Interestingly, transcript levels of some P1 target genes showed higher expression under control conditions in the absence of UV-B in the landraces than in B73, for example *PHENYLALANINE AMMONIA LYASE 2* (*PAL2*, GRMZM2G441347) and *3 (PAL3*, GRMZM2G170692), *UDP-GLUCOSYL TRANSFERASE 1* (*UGT1*, GRMZM2G162755), and *LACCASE 10* (*LAC10;* GRMZM2G140527; Figure 6). In addition, some P1 targets were also highly induced by UV-B in at least one of the landraces, for example *PAL3, FLAVONE SYNTHASE 1* (*FNS1*, GRMZM2G167336), *UGT1*, and *LAC10*. In general, transcript levels of the targets analyzed were higher both before and after the UV-B treatment in the landraces than in B73, with the exception of *FNS1* under control conditions.

This analysis was also carried out simultaneously using RNA samples from other landraces from high altitudes: Cacahuacintle, Conico and Confite Puneño; the results obtained for the expression analysis of these 5 genes were similar to those obtained using samples from Arrocillo and Mishca (Figure S5).

On the other hand, other P1 targets were also analyzed for their expression in high altitude landraces and after UV-B exposure. *CINNAMOYL-COA REDUCTASE* (*CCR*, GRMZM2G068917); *JACALINE 1* (*JAC1*, GRMZM2G314769); *RHAMNOSE SYNTHASE 1* (*RHM1*, GRMZM2G031311); *MULTI ANTIMICROBIAL EXTRUSION PROTEIN* (*MATE*, GRMZM2G079554); *WHITE POLLEN* (*WHP*, GRMZM2G151227); *UDP-GLUCOSYL TRANSFERASE 2* (*UGT2*, GRMZM2G063550) and *4* (*UGT4*, GRMZM2G180283); and *PHENYLALANINE AMMONIA LYASE 1* (PAL1, GRMZM2G334660), which were expressed at significantly higher levels in P1-*rr* compared to P1-*ww* pericarps (Morohashi et al., 2012), did not show a consistent up-regulation by UV-B and higher expression levels in the landraces (Figure S5). Thus, other transcription factors besides P1 may have a major role in the regulation of the expression of this subset of genes in maize leaves.

## Discussion

Solar radiation is an important environmental factor, as it is not only a source of energy for photosynthesis and an informational signal for growth and development, but also contains potentially harmful UV-B radiation (UV-B, 280–315 nm) (Ballare et al., 2001). Although low levels of UV-B can initiate photomorphogenic responses through the activation of the UV-B photoreceptor UVR8 (Nawkar et al., 2013), high UV-B intensities cause cellular damage to proteins, lipids, DNA and RNA molecules (Hollosy, 2002; Gill et al., 2015; Manova and Gruszka, 2015). Plants exposed to UV-B show increased levels of flavonoids, as these compounds are effective UV-B sunscreens and have antioxidant properties (Falcone Ferreyra et al., 2010). On the other hand, chromatin remodeling has also been previously implicated in plant responses to UV-B by several lines of evidence. Transcriptome profiling of high-altitude, UV-B-tolerant maize landraces showed constitutively higher expression of genes predicted to encode chromatin remodeling factors; these landraces also showed UV-B up-regulation of these genes (Casati et al., 2006). Moreover, transgenic plants with decreased expression of four UV-B regulated chromatin factors were hypersensitive to UV-B at doses that do not cause visible damage to normal maize (Casati et al., 2006, 2008; Campi et al., 2012; Questa et al., 2013). In maize, the R2R3-MYB transcription factor P1 controls the accumulation of several UV-B absorbing phenolics by activating a subset of flavonoid biosynthetic genes in leaves of maize landraces adapted to high altitudes. Thus, in this work, we investigated the possible epigenetic mechanisms that could participate in *P1* regulation by UV-B radiation. We here demonstrate that induction of *P1* expression by UV-B correlates with epigenetic changes in specific regions of the gene. These changes occur at the level of DNA methylation of specific sequences in the proximal promoter region, intron 1 and intron 2; as well as by changes in histone methylation and acetylation; and in the levels of smRNAs complementary to *P1* sequences. These modifications take place in both the high altitude landraces Arrocillo and Mishca but also in the low altitude inbred B73. However, the chromatin changes at the *P1* locus by UV-B differ between the lines under study. For example, both under control conditions and after UV-B exposure, the *P1* promoter from the B73 inbred line present a higher degree of methylation than that from the landraces. Interestingly, after UV-B exposure, DNA methylation of B73 *P1* promoter is similar to that of the landraces under control conditions in the absence of UV-B. Thus, *P1* transcription in response to UV-B could be regulated by the DNA methylation state at the proximal promoter. However, intron 1 and 2 methylation state would have a minor role in *P1* expression under UV-B conditions.

We here demostrate that H3K27me3 association to the B73 *P1* promoter is higher than that to Arrocillo and Mishca promoters, both under control conditions and after UV-B exposure. On the other hand, both introns 1 and 2 show a lower H3K9me2 enrichment in the two landraces than in B73 after the UV-B treatment. In addition, both under control conditions and after UV-B exposure, AcH3 enrichment to the *P1* proximal promoter of both landraces is higher than that in B73; and B73 almost duplicates the levels of smRNAs in the landraces both under control conditions and after UV-B exposure. Thus, specific changes at the chromatin structure of the *P1* gene, such as (1) decreased DNA methylation and H3K27me3 at the proximal promoter region, (2) decreased H3K9me2 associated to introns 1 and 2, and (3) decreased smRNA levels complementary to *P1* regions, together with (4) increased H3 acetylation associated with the *P1* promoter, may be important for higher *P1* expression in leaves in high altitude landraces, and its UV-B regulation.

The tissue-specific expression pattern generated by *P1-wr* has previously been attributed to repeat-induced gene silencing through a mechanism that may involve intrachromosomal interactions among repeats (Chopra et al., 1998). However, Sekhon et al. (2007) suggested that distinct regulatory sequences in the P1-*wr* promoter and intron 2 regions could undergo independent epigenetic modifications to generate tissue-specific expression patterns (Sekhon et al., 2007). Although not completely understood, the role of epigenetic mechanisms in regulating the expression of tandem repeated endogenous genes or genes with repeated sequences has been investigated in many systems (Birchler et al., 2000; Chan et al., 2005). In this case, in the presence of a trans-acting modifier Unstable factor for orange1 (Ufo1), *P1-wr* becomes less methylated and shows increased *P1* transcription (Chopra et al., 2003). The Ufo1-induced phenotypes show a range of pigmentation that positively correlates with the degree of demethylation of the *P1-wr* repeat complex. On the other hand, intron 1 contains two RY-like elements. Similar *cis* sequences have been shown to play a role in the regulation of gene expression (Bobb et al., 1997). Generally, regulatory elements are found in the promoter; however, their occurrence in other genic regions has also been documented (Sieburth and Meyerowitz, 1997; Deyholos and Sieburth, 2000; Sheldon et al., 2006). DNA methylation and/or chromatin modifications of the intronic sequences containing regulatory elements may cause transcriptional silencing (Chan et al., 2005; Sheldon et al., 2006). Intronic enhancers have been previously described to regulate the genes they reside within (Mascarenhas et al., 1990; Jeon et al., 2000; Rose, 2002). In particular, it has been proposed that a region of 168-bp in *P1* intron 2 could act as an enhancer, alone or in combination with distal enhancers for the other *P1* copies located downstream (Sekhon et al., 2007).

There are numerous accounts of environmental stresses—such as extreme temperature, drought or ultraviolet radiation—reported to trigger epigenetic changes, thus affecting the transcription certain genes (Matzke and Mosher, 2014). A number of studies have shown that these DNA and histone modifications play a key role in gene expression and plant development under stress conditions (Chinnusamy and Zhu, 2009); however, the precise role for individual chromatin remodeling proteins in the regulation of gene expression in plants is now only being established for a few specific genes. Many of the chromatin-associated factors that have now been characterized in plants mediate posttranslational histone tail modifications or DNA methylation (Wagner, 2003). On the other hand, smRNAs play important regulatory roles in gene expression during development, stress response and phytohormone signaling. In spite of the substantial amount of experimental work with plant smRNAs, little is known about their expression pattern and function during stresses like UV-B. One example is the production of smRNAs by Dicer-like 1 (DCL1) after mechanical wounding in sweet potato. After wounding, DCL1 generates 22 and 24 nt mature smRNAs, and these smRNAs target the first intron of IbMYB1 before RNA splicing, and mediate RNA cleavage triggering the production of secondary smRNAs and DNA methylation of IbMYB1 (Lin et al., 2013). This process finally represses the expression of the IbMYB1 family genes and regulates the phenylpropanoid pathway (Lin et al., 2013). Moreover, studies evaluating the overexpression of *REPRESSOR OF SILENCING 1* (*ROS1*) from Arabidopsis in transgenic tobacco (*Nicotiana tabacum* L.), provided evidence for the epigenetic regulation of genes encoding enzymes of the flavonoid and antioxidant pathways during salt-stress exposure (Bharti et al., 2015).

We here also provide evidence that several P1 target genes in silks and pericarps identified by RNAseq (Morohashi et al., 2012) are also expressed in maize leaves and are UV-B regulated. In particular, three transcripts encoding enzymes of the flavonoid pathway, two *PALs* and one *UGT*, showed higher expression under control conditions in the absence of UV-B in the landraces than in B73. Moreover, some of P1 targets were also highly induced by UV-B in at least one of the high altitude lines, for example *PAL3, FNS1, UGT1*, and *LAC10*. For this set of transcripts, in general expression levels were higher both before and after a UV-B treatment in the landraces than in B73. Despite this, other P1 target genes in silks and pericarps did not show higher expression in leaves of high altitude landraces and/or in response to UV-B. Thus, it is possible that for the expression of this subset of genes in leaves, other transcription factors and/or environmental conditions may have more important regulatory effects.

Together, the results presented here demonstrate that changes in the chromatin state of the *P1* gene in maize leaves can provide an important regulation mechanism for the expression of this transcription factor under UV-B radiation. These changes in *P1* expression may be key to properly regulate the expression of genes in the phenylpropanoid pathway in leaves, to be able to confer plants with better shielding protection against UV-B. The intelligent exploitation of maize resources for plant breeding requires detailed knowledge and quantification of phenotypes differentially represented among the lines. The alleles responsible for these phenotypes could be bred into lower altitude inbred lines as one route to improve UV-B tolerance.

## Author contributions

SR and PC designed the experiments and wrote the paper. SR and JE did the experiments.

## Funding

This research was supported by FONCyT grants PICT 2012-1521 to SR and PICTs 2012-267 and 2013-268 to PC.

### Conflict of interest statement

The authors declare that the research was conducted in the absence of any commercial or financial relationships that could be construed as a potential conflict of interest.
