# Supplementary material for: P1 Epigenetic Regulation in Leaves of High Altitude Maize Landraces: Effect of UV-B Radiation
Source: Front Plant Sci. 2016 Apr 21;7:523. doi: 10.3389/fpls.2016.00523 (PMC4838615; doi:10.3389/fpls.2016.00523)
Supplement: Supplementary file 1 [file Image1.pdf]

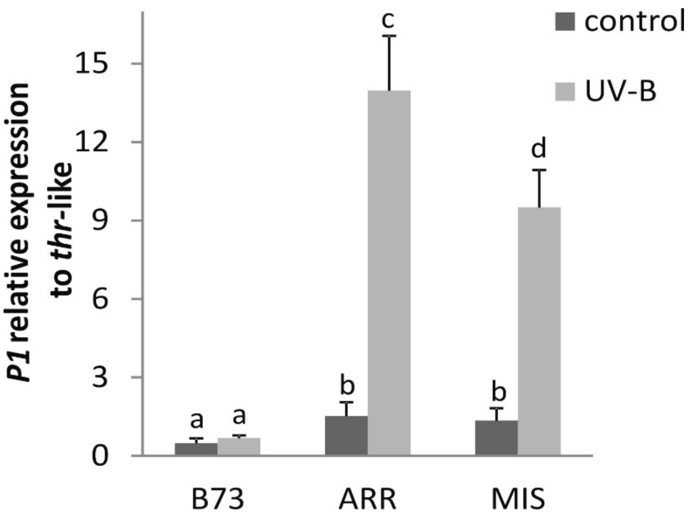

Figure S1. P1 expression analysis under control conditions and after UV-B exposure in leaves of the B73, Arrocillo (ARR) and Mishca (MIS). P1 expression was assayed by qRT-PCR in leaves of B73 and maize landraces ARR and MIS after an 8 h UV-B-treatment and under control conditions in the absence of UV-B. The non-UV-B regulated transcript thioredoxin-like transcript was used for data normalization. Three biological replicates were performed and three qRT-PCR experiments were done with each sample. Error bars are standard errors. Different letters indicate significant differences between control and UV-B condition ($P < 0.05$). EP3-13 and P1-L010 primers sequences are listed in Table 1.
