# Supplementary material for: P1 Epigenetic Regulation in Leaves of High Altitude Maize Landraces: Effect of UV-B Radiation
Source: Front Plant Sci. 2016 Apr 21;7:523. doi: 10.3389/fpls.2016.00523 (PMC4838615; doi:10.3389/fpls.2016.00523)
Supplement: Supplementary file 2 [file Image2.PDF]

Proximal promoter

Primers pair: EP5-8-F/P1007ATG-R

acgcgcgaccagctgctaaccgtgcgcaagtagtagtgcgacttcgccgccggccgggatcgctagctcgatcgatcggcgggaccacatacgactccggtgtgg
ccagcggcggccgggccggggaacgcacgtgctgcgagcgagcgagggcagacgctagctgttgccgggagctagccggcgcgcgatggggagggcgccgt
gctgcga

Intron1

Primers pair: Ex1end-F/EP3-13-R

gtcgctgcccaagaatgcaggtaaaccaaagccggccgcgcgccatgcatcgccacgtagcatcaatctccgatccatgcatatatgagcttcttcttcgtcgccgtcg
tcgttcttagctagttaggacgcgcatgcaggcctgctccggtgcggcaagagctgccggctccggtggatcaactaccttcgggcgga

Intron2

Primers pair: 10908-F/11202 R

gtcctgtccatttcgctttgctgtctcgccatcaccgccggccggtactgtgcaaagcgcgcattggctagcttccctgttgccttgatctgatcatagaggggtccctgtc
cgtggcaaacagtgggattaatgacgggcccacgccggccctccctaatgatttcccccgccttaacgttgacactgcggacggtgagaggcgcggctgtggacgt
acggcgagatctatcgggggcccatcgtccttcgcgaacgatgcgtacatttagcgccatatataaacacacgccg

Figure S2. Sequences amplified by qPCR in ChIP analysis. Each pair of primers sequences is detailed in Table 1. Highlighted sequences correspond to primer sequences and underlined CCGG indicate HpaII restriction sites.
