# Supplementary material for: P1 Epigenetic Regulation in Leaves of High Altitude Maize Landraces: Effect of UV-B Radiation
Source: Front Plant Sci. 2016 Apr 21;7:523. doi: 10.3389/fpls.2016.00523 (PMC4838615; doi:10.3389/fpls.2016.00523)
Supplement: Supplementary file 3 [file Image3.PDF]

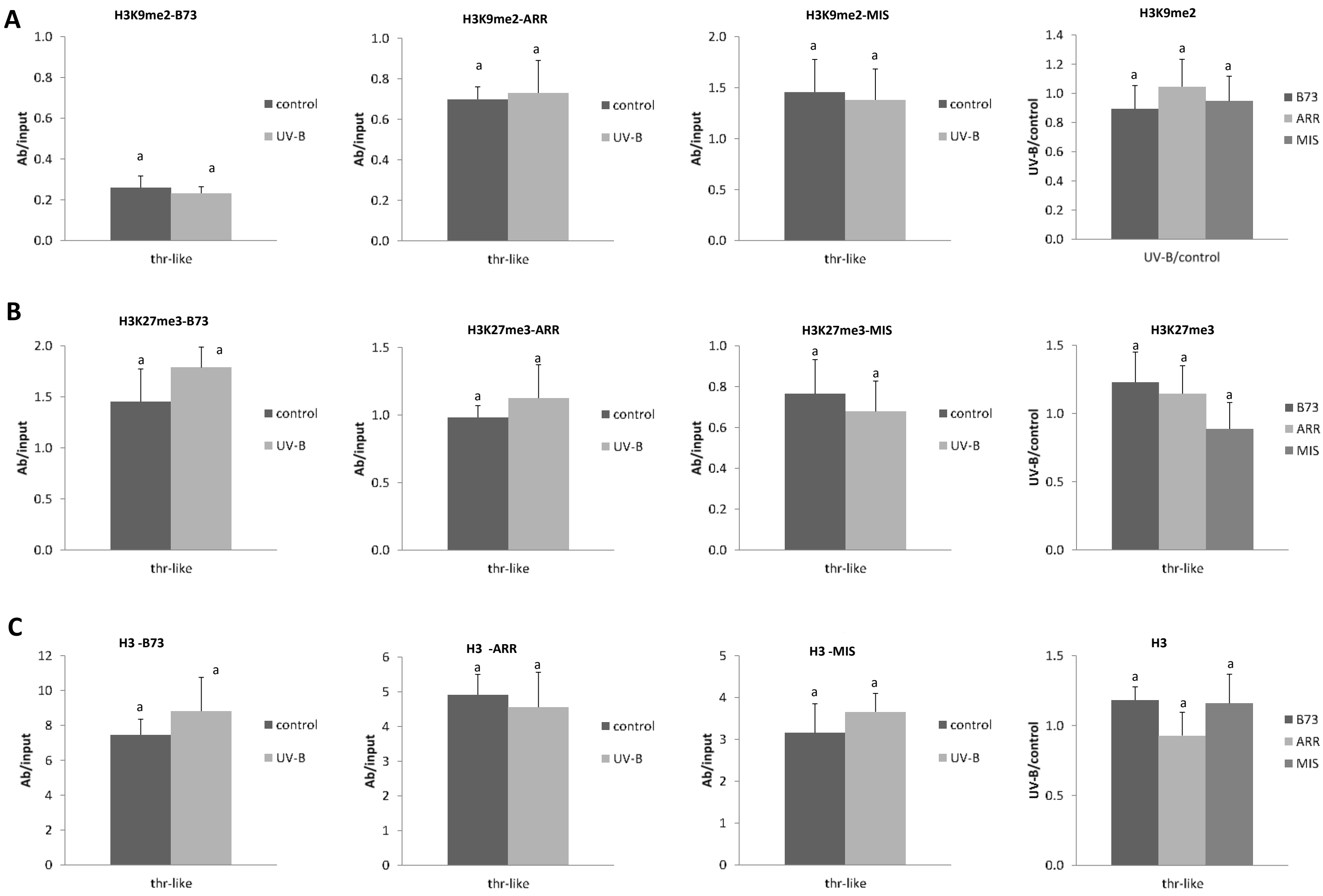

Figure S3. Methylation state of K9 and K27 of H3 associated with a control gene that is not UV-B-regulated (thioredoxin-like; AW927774) by qPCR of B73, Arrocillo (ARR) and Mishca (MIS). ChIP assays were done using antibodies specific for H3K9me2 (A), H3K27me3 (B) or total histone H3 (C) in nuclei prepared from leaves of inbred and landraces plants after a UV-B treatment (UV-B) or under control conditions (control). Enriched fractions from UV-B treated vs. control plants were compared. ChIP data were normalized to input DNA before immunoprecipitation. The signal detected in samples incubated in the absence of any antibody as a control was less than 5% of the signal when antibodies were used. Error bars are standard errors. Statistical significance was analyzed using ANOVA, Tukey test with $P < 0.05$; differences from the control are marked with different letters. Three biological replicates of chromatin immunoprecipitation were used for data normalization.
