# Supplementary material for: P1 Epigenetic Regulation in Leaves of High Altitude Maize Landraces: Effect of UV-B Radiation
Source: Front Plant Sci. 2016 Apr 21;7:523. doi: 10.3389/fpls.2016.00523 (PMC4838615; doi:10.3389/fpls.2016.00523)
Supplement: Supplementary file 4 [file Image4.PDF]

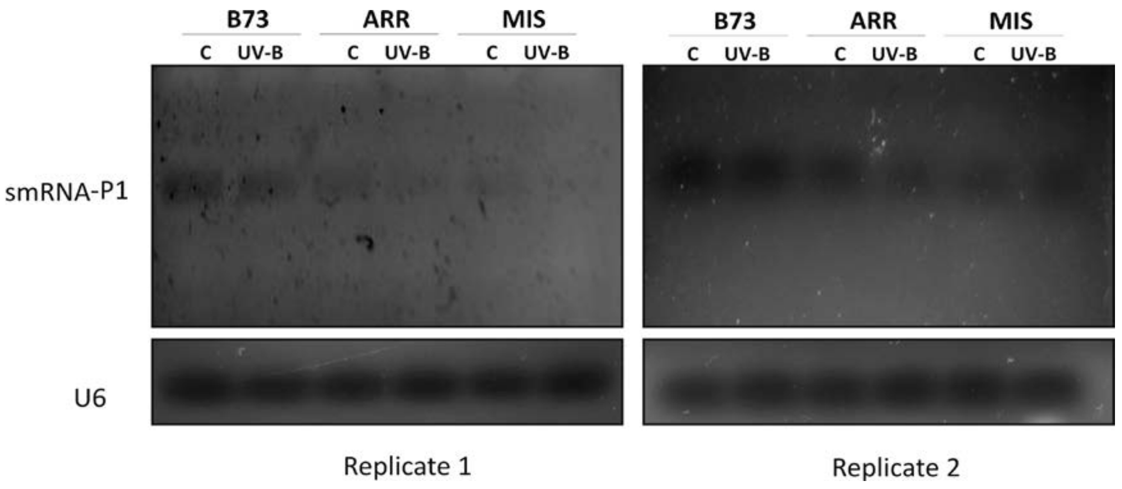

Figure S4. Northern blot replicates for the analysis of the expression of smRNAs complementary to the P1 proximal promoter and the 3' end of intron 2 in leaves of B73, Arrocillo (ARR) and Mishca (MIS). Plants were irradiated during 8 h under UV-B or kept under control conditions in the absence of UV-B. (A) Northern blot analysis developed using 32P-labeled P1 smRNA probes complementary to the P1 proximal promoter (probes 4 and 5) and the 3' end of intron 2 (probe 6), described in Table 3, or alternatively with an specific U6 probe. U6 mRNA was used as a control of equal loading of RNA in each lane. Each blot is representative of three individual experiments.
