# Supplementary material for: P1 Epigenetic Regulation in Leaves of High Altitude Maize Landraces: Effect of UV-B Radiation
Source: Front Plant Sci. 2016 Apr 21;7:523. doi: 10.3389/fpls.2016.00523 (PMC4838615; doi:10.3389/fpls.2016.00523)
Supplement: Supplementary file 5 [file Image5.PDF]

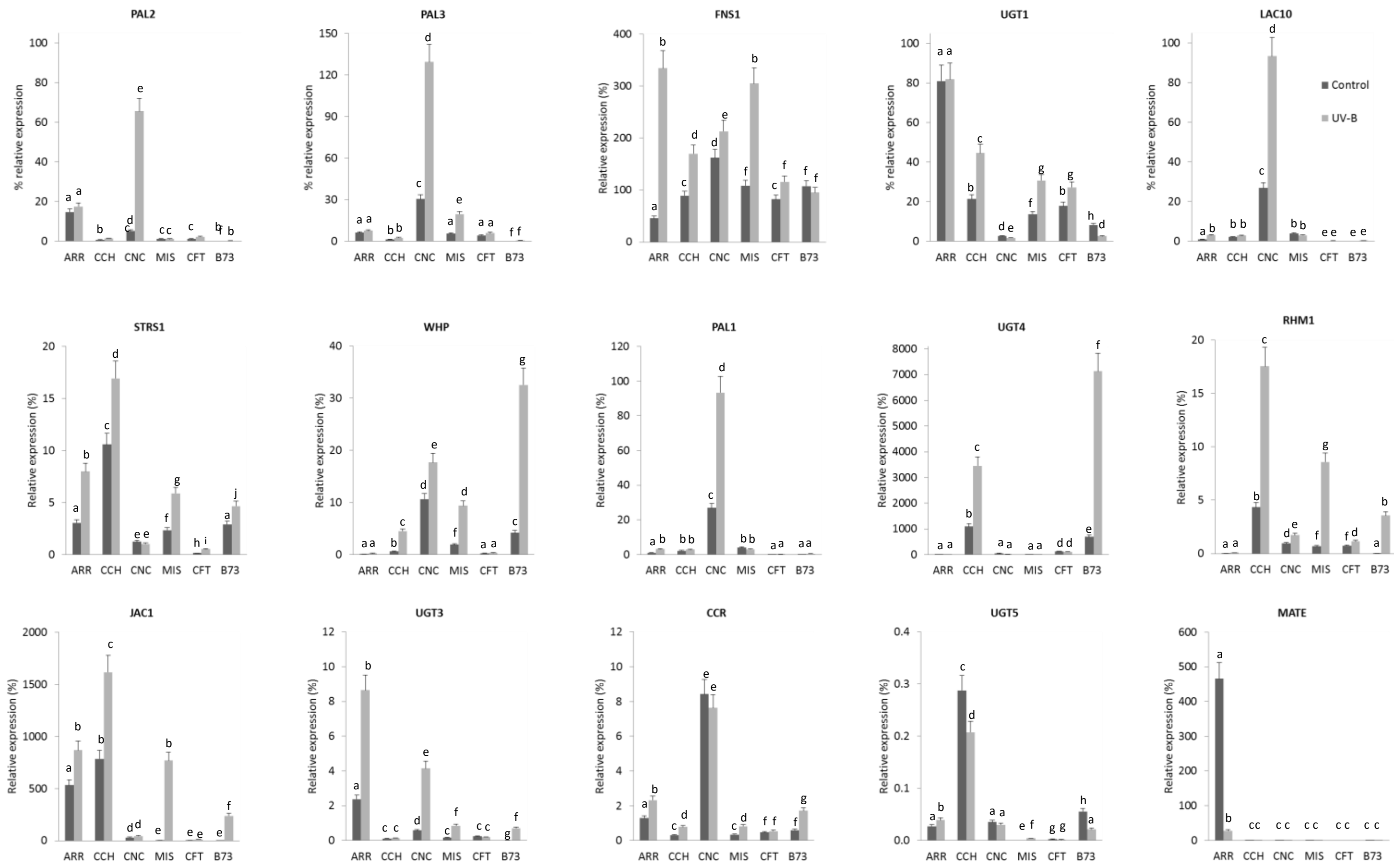

Figure S5. Expression analysis of P1 target genes under control conditions and after UV-B exposure in leaves of the B73 inbred line and high altitude landraces. Fifteen genes that showed higher expression in P1-rr than in P1-ww pericarps and silks by RNA-Seq experiments were chosen for their expression analysis in leaves of five high altitude landraces (Arrocillo (ARR), Cacahuacintle (CCH), Confite Puneño (CFT), Mishca (MIS), Cónico Norteño (CNC)) and the B73 inbred line in response to UV-B or under control conditions in the absence of UV-B. Three biological replicates were performed for each sample plus template-free samples and other negative controls (reaction without reverse transcriptase). Amplification of a thioredoxin-like transcript was used for data normalization. Error bars are standard errors. Different letters indicate significant differences between control and UV-B condition ($P < 0.05$). The transcripts analyzed correspond to: CINNAMOYL-COA REDUCTASE (CCR, GRMZM2G068917); STRICTOSIDINE SYNTHASE (STRS1, GRMZM2G177928); LACCASE (LAC10, GRMZM2G140527); JACALINE (JAC1, GRMZM2G314769); RHAMNOSE SYNTHASE (RHM1, GRMZM2G031311); MULTI ANTIMICROBIAL EXTRUSION PROTEIN (MATE, GRMZM2G079554); WHITE POLLEN (WHP, GRMZM2G151227); UDP-GLUCOSYL TRANSFERASE (UGT1, GRMZM2G162755; UGT2, GRMZM2G063550; UGT4, GRMZM2G180283); PHENYLALANINE AMMONIA LYASE (PAL1, GRMZM2G334660; PAL2, GRMZM2G441347; PAL3, GRMZM2G170692) and FLAVONE SYNTHASE (FNS, GRMZM2G167336).
